# A Case of Acute Pancreatitis in a Patient Receiving High-Dose Steroids for Optic Neuritis

**DOI:** 10.7759/cureus.19132

**Published:** 2021-10-29

**Authors:** Kinza Iqbal, Sawai Singh Rathore, Vinay Hanyalu Shankar, Keerti Deepika, Vishwanath Pattan, Thoyaja Koritala, Nitesh Jain, Ramesh Adhikari

**Affiliations:** 1 Internal Medicine, Dow University of Health Sciences, Karachi, PAK; 2 Internal Medicine, Dr. Sampurnanand Medical College, Jodhpur, IND; 3 Pathophysiology, American University of Antigua, St. George, ATG; 4 Pediatrics/Translational Research, Thomas Jefferson University, Philadelphia, USA; 5 Endocrinology, Wyoming Medical Center, Casper, USA; 6 Internal Medicine, Mayo Clinic, Mankato, USA; 7 Pulmonary and Critical Care Medicine, Mayo Clinic, Mankato, USA; 8 Hospital Medicine, Franciscan Health, Lafayette, USA; 9 Geriatrics, Brown University, Providence, USA

**Keywords:** pancreatitis, severe pancreatitis, acute optic neuritis, steroid induced pancreatitis, drug-induced pancreatitis, acute pancreatitis, steroids, medication-induced pancreatitis

## Abstract

Although rare, drug-induced pancreatitis is an important cause of acute pancreatitis. The diagnosis of drug-induced pancreatitis remains a challenge for clinicians. Steroids are one of the frequently used drugs in hospitals for many acute illnesses. Patients presenting with signs and symptoms of acute pancreatitis, with a recent history of steroid use, in the absence of other potential causes, should be approached with a high suspicion for steroid-induced pancreatitis to ensure a timely diagnosis. We describe a case of a 57-year-old female treated for optic neuritis of the left eye with high doses of Methylprednisolone for five days, who presented to the emergency room with acute abdominal pain within 24 hours of discharge. A detailed evaluation of the patient's medical history and exclusion of other probable etiologies confirmed the diagnosis of steroid-induced pancreatitis. Withdrawal of the offending agent and supportive care resolved the underlying acute pancreatitis.

## Introduction

Acute pancreatitis is characterized by the sudden onset of inflammation of the pancreas; it is marked by symptoms including abdominal pain radiating to the back, fever, nausea, and vomiting [1]. Pancreatitis caused by drugs is relatively infrequent, as the more common causes of pancreatitis include alcohol abuse, hypertriglyceridemia, and gallstones [1,2]. Drug-induced pancreatitis accounts for about 0.1% to 2% of all cases [2]. Its severity varies from patient to patient, but it is often low to moderate in severity and is usually not accompanied by complications [2]. Only a few cases of steroid-induced pancreatitis have been described previously in the literature [3,4]. A comprehensive study showed an increased risk of acute pancreatitis among users of oral glucocorticoids instead of the non-users (OR: 1.53; 95% CI, 1.27-1.84) [5]. The unique part of the study was that the risk was high between 4 and 14 days of glucocorticoid use, while the risk was minimal in the first few days of steroid use. Therefore, it is imperative to establish a definitive diagnosis of steroid-induced pancreatitis based on a detailed evaluation of the patient's medical history and exclusion of other probable etiologies. In this case report, we describe a case of acute pancreatitis induced by steroids in a patient with optic neuritis.

## Case presentation

A 57-year-old Caucasian female with a history of hypertension and cholecystectomy, and a recent diagnosis of optic neuritis in the left eye with impaired vision, who was treated with intravenous steroids (methylprednisolone 1 gram daily for five days) and discharged on a tapering dose of steroids (initial dose: 60 mg prednisone daily), presented to the emergency room less than 24 hours after the discharge for evaluation of abdominal pain with vomiting.

After discharge, the patient experienced pain in the left side of her abdomen and had an episode of non-bloody vomiting. She had no reported diarrhea, black stools, or blood in the stools. The patient took Aleve (ibuprofen) for pain, which did not relieve the pain completely. She denied alcohol consumption. The patient had no fever, chills, cold, cough, shortness of breath, body aches, urinary complaints, loss of sensation of smell, and/or new changes in vision.

Her prescription medications included metoprolol succinate, spironolactone, albuterol inhaler, multivitamins, and the newly started prednisone 60 mg daily with a tapering regimen. On physical examination, she was noted to have mild tenderness in the left hypochondrium and left lumbar regions with no guarding or rigidity. Murphy's sign was negative. No organomegaly was appreciated. The patient had blurred vision in the left eye attributed to her recent optic neuritis with no new changes in her vision, compared to the previous exam done before discharge. The initial lipase level was 1212 U/L (normal range: 11-82 U/L), which eventually became normalized by the time of discharge 55 U/L (normal range: 11-82 U/L). The laboratory values of the patient at the time of admission are presented in Table 1.

**Table 1:** Laboratory values. WBC: white blood cells; RBC: red blood cells; MCV: mean corpuscular volume; MCH: mean corpuscular hemoglobin; MCHC: mean corpuscular hemoglobin concentration; RDW: red cell distribution width; MPV: mean platelet volume; SGOT: serum glutamic-oxaloacetic transaminase; A/G ratio: albumin to globulin ratio; HDL: high-density lipoprotein; VLDL: very-low-density lipoprotein; CHOL HDL-C ratio: total cholesterol to high-density lipoprotein cholesterol ratio; LDL: low-density lipoprotein; mg/dl: milligrams per deciliter; U/L: units per liter; g/dl: grams per deciliter; pg: picograms; fL: femtoliter; mmol/L: millimoles per liter; mEq/L: milliequivalents per liter; µL: microliter.

Laboratory parameter	Patient’s values	Normal values
CBC		
WBC (10^3^/µL)	25.5	4.0 - 11.0
RBC (10^6^/µL)	4.85	3.63 - 5.04
Hemoglobin (g/dL)	14.1	12.0 - 15.3
Hematocrit (%)	41.1	34.7 - 45.1
MCV (fL)	84.9	80.0 - 100.0
MCH (pg)	29.1	26.0 - 34.0
MCHC (g/dL)	34.3	32.5 - 35.8
RDW (%)	13	11.9 - 15.9
Platelets (10^3^/µL)	309	150 - 450
MPV (fL)	7.4	6.8 - 10.2
WBC Differential		
Neutrophil %	71	43.0 - 82.3
Band neutrophil %	1	0.0 - 10
Lymphocyte %	23	14.5 - 45.2
Monocytes %	3	4.3 - 13.3
Eosinophil %	0	0.1 - 6.8
Basophil %	0	0.0 - 2.0
Metamyelocyte	1	0
Chemistries		
Sodium (mmol/L)	127	133 - 144
Potassium (mmol/L)	3.5	3.5 - 5.2
Chloride (mmol/L)	95	98 - 107
Carbon dioxide (mmol/L)	24	21 - 31
Anion gap (meq/L)	8	6.2 - 14.7
Blood urea nitrogen (mg/dl)	26	7 - 25
Creatinine (mg/dl)	0.7	0.6 - 1.2
Calcium (mg/dl)	8.7	8.6 - 10.3
Glucose (mg/dl)	247	70 - 99
Total Alkaline phosphatase (U/L)	98	34 - 104
Total protein (g/dl)	7.1	6.4 - 8.9
Albumin (g/dl)	3.7	3.5- 5.7
Aspartate transaminase (SGOT) (U/L)	17	13 - 39
Alanine transaminase (U/L)	28	7 - 52
A/G ratio	1.09	0.76 - 1.76
Total bilirubin (mg/dl)	0.9	0.0 - 1.0
Lipase (U/L)	1,212	11 - 82
Lipid profile		
Total cholesterol (mg/dl)	163	< 200
Triglycerides (mg/dl)	214	< 150
HDL cholesterol (mg/dl)	49	> 40
CHOL HDL-C ratio	3.3	≤ 5
VLDL (mg/dl)	43	5 - 30
Non-HDL cholesterol (mg/dl)	114	< 130
LDL cholesterol (mg/dl)	71	0 - 129

**Figure 1:**
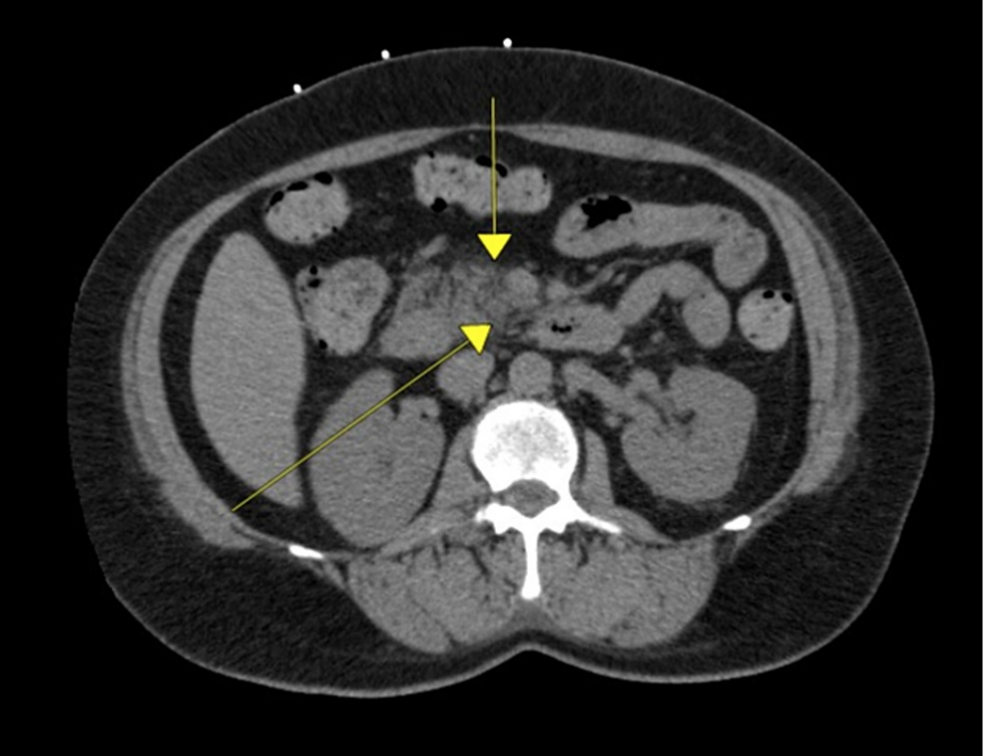
Computed tomography of abdomen and pelvis without contrast showing infiltrative/inflammatory change of fat around the pancreas.

Computed tomography (CT) of abdomen and pelvis without contrast (Figure 1) showed infiltrative/inflammatory change of fat around the pancreas, second and third portion of the duodenum, and to a lesser degree in the left perinephric space. Findings were consistent with pancreatitis, duodenitis, and duodenal ulcer disease with no evidence of perforation or left pyelonephritis. No fluid collections were observed.

An ultrasound of the abdomen showed hepatic steatosis with hepatomegaly and post-cholecystectomy changes with no common bile duct stone. A slightly prominent pancreas was also noted, likely related to acute pancreatitis. A gastroenterologist was consulted for further evaluation and an esophagogastroduodenoscopy (EGD) was performed due to the abnormal CT abdomen and pelvis findings. EGD findings included a 1-cm hiatal hernia with a normal duodenum. Los Angeles (LA) Grade B esophagitis and erythematous gastric mucosa were also noted.

COVID-19 polymerase chain reaction (PCR) pre-procedure testing was negative. Cancer antigen 19 (CA 19-9) was 78.0 U/mL (normal ≤ 35 U/mL). Magnetic resonance imaging (MRI) of the abdomen with and without contrast was performed due to elevated CA 19-9. It showed mild heterogeneous hepatic steatosis and mild hepatomegaly. Ill-defined margins of the pancreas with tiny scattered cystic changes were noted, related to pancreatitis.

With no other identifiable cause of acute pancreatitis, it was concluded that the acute pancreatitis was most likely due to steroid use. Steroids were stopped after consulting the neurologist who prescribed the steroids and supportive care. The patient was treated with intravenous (IV) fluids and analgesics. She improved clinically during the hospital stay and was able to tolerate a soft diet by the time of discharge with no abdominal pain, nausea, or vomiting. The neurologist recommended no further continuation of oral steroids. At the time of release, the patient had no additional worsening of visual symptoms. The patient was advised to avoid steroid use and recommended to follow up with a gastroenterologist as an outpatient with a repeat CA 19-9.

## Discussion

Drug-induced acute pancreatitis is a rare occurrence, accounting for 0.5%-2% of all cases [3]. The current knowledge of drug-induced pancreatitis is largely based on evidence from case reports. As many drugs are linked with the development of drug-induced pancreatitis, the implicated drugs are categorized into four classes (Table 2) based on their likelihood to be established as a cause of acute pancreatitis [6]. Steroid-induced pancreatitis has been previously reported in the literature and methylprednisolone, the steroid used in our case, has already been identified as a class one A drug [6]. The mechanism by which steroids affect the pancreas is poorly understood, however, it is suggested that steroids increase the viscosity of pancreatic secretions and delay their emptying [4]. Owing to the significant number of deaths reported in the patients with steroid-induced acute pancreatitis, this class of drugs is thought to cause a severe disease course [7].

**Table 2:** Drug classification system for assessment of the likelihood of drug-induced pancreatitis. N/A: not available; *: consistent latency defined as >75% of cases falling into the same latency category (category 1: < 24 hours, category 2: 1–30 days, and category 3: > 30 days).

Drug class	Minimum number of case reports in humans	Requirement of positive re-challenge	Latency	Other causes of pancreatitis ruled out	Other requirements
Ia	1	Yes	N/A	Yes	-
Ib	1	Yes	N/A	No	-
Ic	1	No	N/A	No	-
II	2	No	Consistent*	No	-
III	2	No	Inconsistent*	No	-
IV	1	N/A	N/A	N/A	Drugs not fitting into the other three classes

Ataallah et al. reported the case of a 20-year-old man who developed steroid-induced pancreatitis after being treated with intravenous Dexamethasone during hospitalization and oral prednisone after discharge for idiopathic immune purpura [3]. Similarly, Minupuri et al. presented a 61-year-old female who developed acute pancreatitis on two different occasions, both occurring after steroid exposure (prednisone 10 mg/day) [4]. In concordance with our findings, Ataallah et al. and Minupuri et al. diagnosed steroid-induced pancreatitis based on the recent history of steroid use after ruling out other potential causes of acute pancreatitis. Discontinuation of steroids and supportive care resulted in clinical improvement with the resolution of pancreatitis [3,4].

Owing to the unsuspected nature of drug-induced pancreatitis and the extensive workup required to rule out other possible causes of acute pancreatitis, the diagnosis of drug-induced pancreatitis remains a challenge for clinicians [3,7]. A timely diagnosis is imperative to avoid complications, including pancreatic necrosis and infection, pancreatic pseudocyst, chronic pancreatitis, and multiorgan failure [4]. A thorough review of the patients’ medications is important in all cases of acute pancreatitis. A clear history of recent steroid use, in the absence of other causes of acute pancreatitis, warrants immediate cessation of steroids to prevent further pancreatic damage [8].

## Conclusions

Although rare, drug-induced pancreatitis is an important etiology of acute pancreatitis. Steroids are commonly used in hospitals for many acute illnesses. For a timely diagnosis of steroid-induced pancreatitis, it is crucial to consider the possibility of drug-induced pancreatitis in a patient presenting with signs and symptoms of acute pancreatitis, with a history of recent steroid use, in the absence of other probable etiologies. Discontinuation of the steroids and supportive care usually resolve the underlying acute pancreatitis and improve the patient's condition.
